# Complete Genome Sequence of an Australian Strain of the Lichen-Forming Fungus Endocarpon pusillum (Hedwig)

**DOI:** 10.1128/MRA.01079-20

**Published:** 2020-12-10

**Authors:** Oliver L. Mead, Cécile Gueidan

**Affiliations:** a Australian National Herbarium, National Research Collections of Australia, Commonwealth Scientific and Industrial Research Organization, Canberra, Australia; University of California, Riverside

## Abstract

The cosmopolitan lichen-forming fungus Endocarpon pusillum (Hedwig) has previously been used as a model for the study of symbiosis and drought resistance. Here, we present the annotated genome of the Australian strain *Endocarpon pusillum* EPUS1.4. This genome sequence provides additional information on the ability of this species to produce secondary metabolites.

## ANNOUNCEMENT

Lichens are natural reservoirs of novel compounds, with over 800 lichen compounds described to date (1) and many being used in industry (2). As such, they represent fertile opportunities for bioprospecting. However, lichen-forming fungi are notoriously slow growing and difficult to cultivate under laboratory conditions, and often the symbionts are recalcitrant to isolation (3). To overcome these problems, several lichen metagenomes and genomes from axenic cultures have been sequenced (4, 5). These projects aim to identify the biosynthetic gene clusters responsible for producing valuable molecules. Similarly, to discover novel molecules through genome mining, we sequenced the genome of the model soil crust lichen Endocarpon pusillum (Hedwig). Two other *Endocarpon* genome sequences have recently been reported (5, 6). Park et al. (6) employed a genomics approach to investigate desiccation resistance in a Korean isolate of *E. pusillum*. Wang et al. (5) used genomics and transcriptomics to identify the molecular mechanisms underlying lichen symbiosis. To strengthen the emerging community of lichen genomics, we contribute the annotated genome sequence of an Australian isolate of *E. pusillum*, strain EPUS1.4.

The specimen of *E. pusillum* (C. Gueidan 2364) was collected from the CSIRO Black Mountain site, north of Christian Road in Canberra, Australia, in 2016 and deposited in the CANB collection (accession number CANB 913709). Ascospores were shot onto peptone-dextrose agar (PDA) plates, and single ascospores were isolated and grown on liquid potato-dextrose broth (PD) medium in an incubator with 20°C/18°C 12-h day/night cycles. Before extraction, the stock culture was ground and an inoculate transferred to stationary YSSG medium, consisting of yeast extract (5 g/liter), sucrose (10 g/liter), sorbitol (10 g/liter), and γ-aminobutyric acid (GABA) medium (1 g/liter).

Genomic DNA and total RNA were extracted separately from ca. 20 mg of *E. pusillum* EPUS1.4 using a phenol-chloroform and sodium dodecyl sulfate (2% wt/vol)-beta-mercaptoethanol (1% vol/vol) emulsion at room temperature (7). The John Curtin School of Medical Research generated 25 million paired-end 2 × 300-bp reads from 100 ng genomic DNA on the Illumina MiSeq platform and 150 million paired-end 2 × 75-bp reads from 100 ng RNA on the Illumina NextSeq 500 platform. The Illumina Nextera XT v3 library kit was used to prepare both nucleic acids for sequencing. The Oxford Nanopore (ONP) MinION (FLO-MIN106D, R9) platform generated 1.6 Gb raw reads from 200 ng DNA using a PCR sequencing kit (SQK-PSK004, ONP, UK).

Fastp v0.19.6 (8) was used to trim and describe the quality of all short reads, with default settings, generating 18 million DNA and 144 million RNA high-quality reads. Long-read data were base called and quality controlled using GUPPY v3.2.2-GPU (ONP) (9), generating 400,000 high-quality reads, as counted by NanoPlot (10).

SPAdes v3.12.0 (11), using a kmer length of 127 bp, 20 threads, 256 Gb of RAM, and the--nanopore switch, was used to assemble high-quality genomic DNA (gDNA) short and long reads into a hybrid assembly, EP01v1.6.4. This 33.7-Mb assembly contained 2,902 contigs and had an *N*_50_ value of 158 kb and a GC content of 48.4% (QUAST v4.3 [12]). It contained 3,474 of 4,046 (85.9%) Eurotiomycetes benchmarking universal single-copy ortholog (BUSCO) genes (BUSCO v4.0.1 [13]). Trinity v2.3.2, with default settings, was used to assemble a 126-Mb transcriptome from the RNAseq reads (14). HiSat2 v2.1.0 was used to map these reads to EP01v1.6.4 (15, 16). Funannotate v1.7.1 (17), using the clean, sort, mask, predict, and annotate tools, and Blast2Go v5.2.5 (18), using default settings, annotated EP01v1.6.4. This annotation included *ab initio* gene models from Augustus v3.3.2 (19), derived using --singlestrand=true --cds=on --codingseq=on switches, and the Aspergillus nidulans model, as well as aligned RNAseq reads and transcriptomic evidence, to produce 12,503 predicted gene models.

### Data availability.

Data are available in GenBank under BioProject accession number PRJNA589713 and accession number JAACFV000000000.
